# Oxidative Stress and Inflammation in Uterine–Vascular Adaptation During Pregnancy

**DOI:** 10.3390/antiox14091051

**Published:** 2025-08-26

**Authors:** Maurizio Mandalà

**Affiliations:** 1 Department of Biology, Ecology & Earth Sciences, University of Calabria, 87036 Rende, Italy; m.mandala@unical.it; 2 Department of Obstetrics, Gynecology and Reproductive Sciences, University of Vermont Larner College of Medicine, Burlington, VT 05405, USA

**Keywords:** redox signaling, preeclampsia, IUGR, uterine circulation

## Abstract

During pregnancy, uterine circulation undergoes profound structural and functional adaptations to accommodate the dramatically increased metabolic demands of the growing fetus. Oxidative stress (OS) and inflammation have emerged as central regulators both physiologically, to drive vascular remodeling and angiogenesis, and pathologically, when dysregulated, to promote endothelial dysfunction, maladaptive extracellular matrix (ECM) remodeling, and heightened arterial stiffness. This review synthesizes insights into the molecular sources of reactive oxygen species (ROS) in the uterine vasculature, endothelial and immune-mediated inflammatory pathways, the bidirectional crosstalk between OS and inflammation, and their combined impact on vascular stiffness. We further discuss clinical implications for conditions such as preeclampsia and intrauterine growth restriction (IUGR), highlight circulating and imaging biomarkers of redox–inflammatory imbalance, and evaluate antioxidant and anti-inflammatory therapeutic strategies. Finally, we identify critical knowledge gaps and propose future research directions aimed at translating mechanistic understanding into personalized maternal–fetal care. For this narrative review, we searched the PubMed and Web of Science databases to identify all human and animal studies investigating OS and inflammation on uterine vasculature remodeling during pregnancy.

## 1. Introduction

Pregnancy imposes one of the most dramatic cardiovascular challenges encountered in *human* physiology: uterine blood flow rises almost twelve-fold, from ~50 mL min^−1^ in the non-pregnant state to >700 mL min^−1^ at term [1]. To accommodate this surge, maternal uterine resistance arteries undergo outward hypertrophic remodeling; trophoblasts transform spiral arteries into high-capacity, low-resistance conduits; and vascular tone is re-programmed in favor of sustained vasodilatation [2]. Although nitric-oxide (NO) signaling and angiogenic growth factors [Vascular endothelial growth factor A (VEGF-A), Placental growth factor (PlGF), Angiopoietin 1-2 (Ang-1/2)] have long been recognized as key drivers of these changes, growing evidence shows that ROS and inflammatory signals are also indispensable messengers, coordinating endothelial–smooth-muscle crosstalk, ECM turnover, and immune–trophoblast dialogue [3]. In normal pregnancy, low-to-moderate levels of ROS, mainly generated by nicotinamide adenine dinucleotide phosphate (NADPH) oxidase isoforms (NOX1/4) and mitochondria, activate redox-sensitive kinases and transcription factors [e.g., Nuclear Factor Erythroid 2-Like (NRF2) and Hypoxia-Inducible Factor 1-alpha (HIF-1α)]. This, in turn, enhances VEGF signaling, promotes smooth muscle cell (SMC) phenotypic switching, and facilitates matrix-metalloproteinase (MMP) activation [4,5]. Loss of this delicate equilibrium underlies the pathogenesis of major obstetric complications. Excessive ROS, from exaggerated NADPH oxidase (NOX) activity, mitochondrial dysfunction, or xanthine oxidase conversion, quench NO, uncouple endothelial NO synthase (eNOS), and generate peroxynitrite (ONOO^−^), leading to endothelial dysfunction, maladaptive collagen deposition and arterial stiffening [6,7]. Concurrent chronic low-grade inflammation promotes leukocyte adhesion, activation of the NOD-like receptor family pyrin domain-containing 3 (NLRP3)-inflammasome, and the release of anti-angiogenic factors [soluble fms-like tyrosine kinase-1 (sFlt-1) and soluble endoglin (sEng)], culminating in the inadequate spiral artery transformation, placental hypoxia, and systemic hypertension that characterize preeclampsia and IUGR [8]. Notably, circulating redox–inflammatory biomarkers (isoprostanes, 3-nitrotyrosine, uric acid) and Doppler-derived uterine-artery pulsatility indices provide clinically accessible windows into this dysregulated milieu and correlate with adverse perinatal outcomes [9], Table S1. This review therefore synthesizes mechanistic and translational advances linking oxidative stress and inflammation to uterine–vascular adaptation. We dissect the enzymatic and mitochondrial sources of ROS; delineate immune-mediated inflammatory cascades and their interplay with redox signaling; examine how redox–inflammatory imbalance promotes vascular stiffness and pregnancy disease; and appraise emerging diagnostic biomarkers and targeted antioxidant/anti-inflammatory strategies. By integrating *human* and animal data, we aim to identify knowledge gaps and foster precision interventions that safeguard maternal–fetal vascular health.

## 2. Physiology of Uterine–Vascular Adaptation

**Figure 1:**
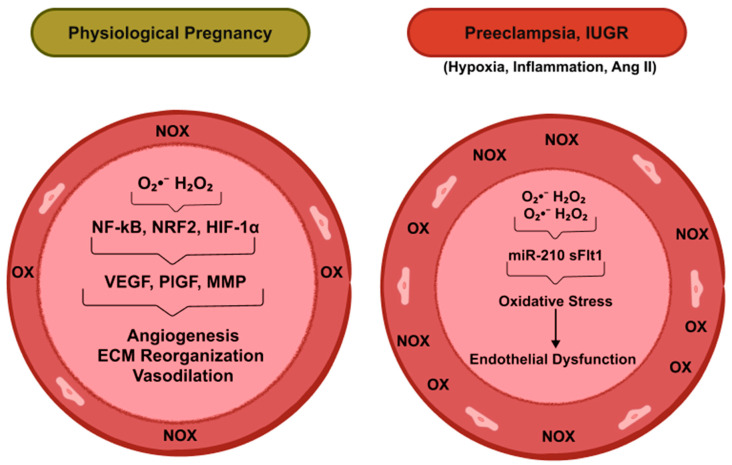
Schematic cross-sections of uterine arteries illustrating reactive oxygen species (ROS) generation and its impact on vascular remodeling during pregnancy. Left panel: Physiological pregnancy: Moderate ROS production originating from mitochondrial respiration, NADPH oxidases, and xanthine oxidase activates redox-sensitive transcription factors (NF-κB, Nrf2, HIF-1α). These, in turn, upregulate pro-angiogenic and matrix-remodeling mediators (VEGF, PlGF, MMPs), driving angiogenesis, extracellular-matrix reorganization, and vasodilation. The net result is outward hypertrophic remodeling, marked by a wider lumen without a change in wall thickness. Right panel: Pathological pregnancy (preeclampsia, IUGR): Chronic hypoxia, elevated angiotensin II (Ang II), and inflammatory cytokines induce mitochondrial dysfunction and excessive ROS accumulation. Via the miR-210–sFlt-1 signaling pathway, this oxidative stress triggers endothelial dysfunction and vascular injury, leading to inward eutrophic remodeling, characterized by a narrowed lumen and increased wall thickness. Hydrogen peroxide (H_2_O_2_); Hypoxia-Inducible Factor 1-alpha (HIF-1α); Matrix-metalloproteinase (MMP); MicroRNA-210 (miR-210); Mitochondria (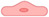); NADPH oxidase (NOX); Nuclear Factor Erythroid 2-Like 2 (NRF2); Nuclear factor-kappa B (NF-κB); Placental growth factor (PlGF); Superoxide (O_2_^•−^); Soluble fms-like tyrosine kinase-1 (sFlt-1); Vascular endothelial growth factor (VEGF); Xanthine oxidase (XO).

To accommodate the significant increase in uteroplacental blood flow in pregnancy, the entire maternal uterine circulation must grow considerably. Many studies have shown that uterine arteries and veins increasing in length and in diameter undergo outward (expansive) hypertrophic remodeling [10]. Several mechanisms have been implicated in the uterine vascular remodeling process. At the deciduo–myometrial interface, extravillous trophoblasts invade spiral arteries from 8 weeks gestation, displacing endothelial cells (ECs) and SMCs and transforming them into high-capacity, low-resistance conduits devoid of maternal vasomotor control [11]. Pregnancy simultaneously re-programs uterine vascular tone and shifts the balance between EC vasodilators and vasoconstrictors toward vasodilation by upregulating eNOS expression and activity, downregulating endothelin-1 (ET-1) release, and enhancing endothelium-derived hyperpolarizing factor (EDHF)-mediated SMC hyperpolarization [12]. SMCs undergo a phenotypic transition from a contractile to a synthetic phenotype, characterized by increased MMP secretion and reduced expression of contractile proteins (SM-α-actin, SM-myosin heavy chain). MMPs degrade collagen I/III and elastin fibers, while tissue inhibitor of metalloproteinase (TIMP) levels rise to prevent excessive matrix loss [13]. Proper ECM remodeling is essential for maintaining vessel compliance and allowing progressive diameter expansion throughout gestation [14]. The placental and decidual angiogenic factors VEGF-A, PlGF, and angiopoietin 1/2 synergize with NO to drive endothelial proliferation, SMC migration and MMP activation [15]. All of these processes are tuned by a physiological redox and low-grade inflammatory milieu, Figure 1. Moderate ROS levels potentiate eNOS phosphorylation, amplify VEGF signaling, and activate redox-sensitive MMPs promoters [4]. When oxidative or inflammatory balance is lost as in preeclampsia, gestational hypertension, and IUGR, NO bioavailability falls, anti-angiogenic factors (sFlt-1, sEng) rise, trophoblast invasion is shallow, and spiral arteries remain high-resistance, with impaired uterine vascular remodeling culminating in placental hypoxia and clinical disease [4,16].

## 3. Origin of Reactive Oxygen Species in the Uterine Vasculature

### 3.1. NADPH Oxidase Family (NOX1–5)

The NOX family constitutes the predominant enzymatic source of ROS in vascular cells, playing a crucial role in redox signaling and oxidative stress-related vascular remodeling during pregnancy [17]. In the uterine vasculature, particularly in ECs and SMCs, NOX1 and NOX4 are constitutively expressed, while NOX2 and NOX5 expression is more dynamic and inducible under pathophysiological stimuli such as hypoxia, angiotensin II (Ang II), and inflammation [18,19]. Upon activation, NOX enzymes transfer electrons from cytosolic NADPH across biological membranes to reduce molecular oxygen to superoxide (O_2_^•−^). The enzymatic activity of NOX1–3 typically requires assembly with regulatory subunits including p22^phox, p47^phox, p67^phox, and Rac1 [20,21]. NOX4, in contrast, is constitutively active and primarily generates hydrogen peroxide (H_2_O_2_) rather than O_2_^•−^, owing to its unique C-terminal domain and subcellular localization in mitochondria and the endoplasmic reticulum [22]. Vascular cells have evolved to use ROS, such as O_2_^•−^ and H_2_O_2_, as signaling molecules [23]. Under physiological conditions, low-to-moderate levels of ROS contribute to uterine vascular remodeling by promoting angiogenesis, ECM reorganization, and vasodilation via activation of redox-sensitive transcription factors such as NRF2 and HIF-1α [24,25,26]. However, dysregulation of NOX enzymes under pathological conditions leads to excessive ROS production, resulting in oxidative stress and endothelial dysfunction. Such processes are implicated in several vascular dysfunctions, including pulmonary hypertension and preeclampsia [27,28]. In preeclampsia, upregulation of NOX2 is consistently observed in uteroplacental vessels and circulating leukocytes, correlating with elevated O_2_^•−^ levels, impaired NO bioavailability, and heightened vascular resistance [10]. Moreover, NOX5, which is regulated by intracellular calcium rather than cytosolic subunits, has been implicated in *human* placental vascular dysfunction, although its role in uterine vasculature remains underexplored due to its absence in rodents [29]. Recent transcriptomic analyses suggest NOX5 upregulation in ECs from preeclamptic placentas, linking calcium-dependent oxidative bursts to inflammation and endothelial injury [29]. Animal models further support the pathological relevance of NOX isoforms. In pregnant sheep, exposed to long-term high altitude, hypoxia increased uterine arterial NOX2 protein expression and enhanced the ROS production associated with suppression of pregnancy-induced upregulation of ryanodine receptor (RyR)-induced Ca^2+^ sparks and BK_Ca_ channel activity [26,30]. This results in elevated myogenic reactivity and impaired uterine vascular adaptation factor, known to increase the risk of preeclampsia and IUGR. Notably, high-altitude hypoxia induces upregulation of miR-210, which may mediate these maladaptive responses [31]. Moreover, plasma from preeclamptic women can upregulate NOX2 and downregulate KCa3.1 expression in umbilical vessels and HUVECs, further contributing to endothelial dysfunction and vascular pathology [16,32]. The important role of NOX in uterine vasculature has been confirmed by knockout studies that have demonstrated that NOX2-deficient rats exhibit reduced uterine arterial resistance and improved fetal outcomes under preeclamptic conditions [33]. Additionally, pharmacological inhibition of NOX1/2 with GKT137831 mitigates oxidative damage and restores vascular function [34]. A recent clinical study has demonstrated a stronger positive correlation between NOX activity and the uterine artery pulsatility index (UtA-PI), suggesting that excessive ROS production contributes to elevated UtA impedance, a common marker of adverse pregnancy outcomes [9]. Overall, the NOX family serves as a critical interface between physiological ROS signaling and pathological oxidative stress in the uterine vasculature. Targeting specific NOX isoforms may offer therapeutic potential in managing pregnancy complications associated with oxidative stress such as preeclampsia and IUGR.

### 3.2. Mitochondria

Mitochondria represent a key intracellular source of ROS, primarily generated at complexes I and III of the electron transport chain through electron leakage. This leads to the production of O_2_^•−^ and, subsequently, H_2_O_2_ via the action of mitochondrial superoxide dismutase (MnSOD). Mitochondrial ROS (mtROS) are not merely byproducts of cellular respiration but play a pivotal modulatory role in signaling cascades that govern uterine vascular remodeling during pregnancy. mtROS regulate MMP activity, particularly MMP-2 and MMP-9, facilitating ECM breakdown and structural adaptation of the uterine vasculature to accommodate increased uteroplacental blood flow [35]. Conversely, pathological conditions such as preeclampsia or IUGR are often characterized by mitochondrial dysfunction and excessive oxidative stress correlates with increased vasoconstriction, arterial wall thickness, and vascular stiffness, ultimately impairing uterine artery remodeling [36,37]. Chronic hypoxia, frequently present in preeclampsia and IUGR, exacerbates the dysfunction by further enhancing mtROS levels [38]. This leads to oxidative damage of lipids, proteins, and mitochondrial DNA, along with the depletion of key antioxidant defenses such as MnSOD and glutathione peroxidase [39]. Evidence from a pregnant sheep model indicates that hypoxia downregulates ten-eleven translocation methylcytosine dioxygenase2 TET (TET2) in uterine arteries, a downstream target of microRNA-210 which impairs mitochondrial bioenergetics and increases mtROS production [40]. Further, in trophoblasts, mtROS activate HIF-1α-dependent microRNA-210, which suppresses iron–sulfur cluster scaffold proteins (ISCU), impairing mitochondrial respiration and promoting apoptosis, thereby inhibiting proper spiral artery remodeling and elevating uterine vascular resistance [41]. The resulting reduction in NO bioavailability, and increased release of antiangiogenic factors such as sFlt-1, amplifies maternal endothelial dysfunction and fetal hypoxia [42]. Moreover, recent studies have shown that, in preeclamptic animal models, CD4+ T cells activate natural killer (NK) cells, exacerbating mitochondrial oxidative stress through immune-mediated mechanisms [43,44]. Preclinical data indicate that administration of mitochondria-targeted antioxidants such as MitoQ reduces oxidative damage, restores uterine artery structure and function, and improves fetal growth outcomes [45]. However, clinical trials with non-targeted antioxidants have yielded limited results, likely due to insufficient mitochondrial bioavailability, underlining the importance of targeted delivery systems in future therapeutic approaches [46]. Elucidating the dualistic nature of mtROS as both signaling molecules under physiological conditions and as potential mediators of vascular dysfunction in pathological states may offer novel insights for the development of redox-based therapeutic interventions aimed at promoting healthy uterine vascular adaptation and improving pregnancy outcomes.

### 3.3. Xanthine Oxidase (XO)

Xanthine oxidoreductase enzyme exists in two interconvertible forms: xanthine dehydrogenase (XDH) and xanthine oxidase (XO). Both isoforms catalyze the oxidation of hypoxanthine to xanthine and subsequently to uric acid, producing O_2_^•−^ and H_2_O_2_ as byproducts. During normal pregnancy, XO activity is tightly regulated and contributes modestly to physiological redox signaling, which is essential for modulating NO bioavailability and supporting angiogenesis and vasodilation within the uterine vasculature [47]. However, under pathological conditions such as preeclampsia and IUGR, XO becomes a prominent source of oxidative stress. Hypoxia, systemic inflammation, and placental ischemia, hallmarks of these pregnancy complications, Table S2, enhance the conversion of XDH to XO in uteroplacental tissues, leading to elevated ROS production [48]. Increased XO activity has been documented in the placenta, maternal serum, and uterine arteries of preeclamptic patients, correlating with elevated serum uric acid levels and vascular dysfunction [49]. Elevations in circulating uric acid in preeclamptic women contribute to the pathogenesis of the disorder, in part, through attenuation of normal trophoblast invasion and spiral artery vascular remodeling [27]. Mechanistically, XO-derived ROS impair endothelial NO signaling, promoting vasoconstriction and reducing uteroplacental perfusion. Furthermore, ROS from XO trigger lipid peroxidation, mitochondrial dysfunction, and apoptosis in endothelial and trophoblastic cells, leading to defective spiral artery remodeling [35]. These alterations promote the release of antiangiogenic factors such as sFlt-1, contributing to increased vascular resistance, maternal hypertension, and compromised fetal oxygenation [37]. From a therapeutic perspective, XO inhibitors, such as allopurinol and febuxostat, have shown antioxidant and vasoprotective effects in cardiovascular and renal pathologies. Preclinical studies suggest that XO inhibition could mitigate oxidative damage in pregnancy-related vascular disorders. For example, allopurinol administration in hypertensive *rat* models reduced placental oxidative stress and improved endothelial function [38]. However, evidence from *human* pregnancy remains limited, and concerns about fetal safety and placental transfer require further investigation [39].

## 4. Antioxidant Systems

### 4.1. Enzymatic Antioxidants

**Figure 2:**
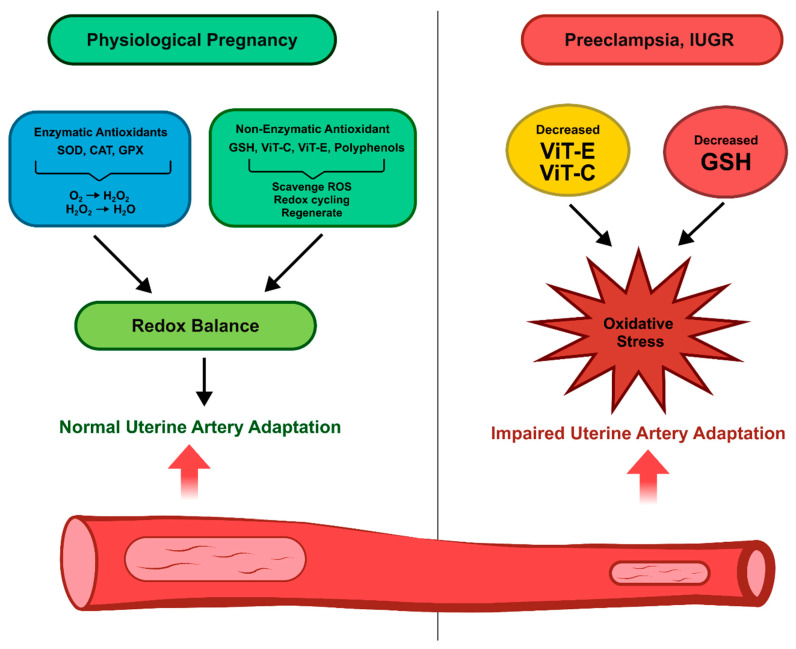
Antioxidant systems and redox balance in the uterine vasculature during pregnancy. The figure illustrates the interplay between enzymatic antioxidants (superoxide dismutase [SOD], catalase [CAT], and glutathione peroxidase [GPx]) and non-enzymatic antioxidants (glutathione [GSH], vitamins C and E) in maintaining physiological ROS levels and promoting uterine vascular remodeling. Impaired antioxidant defense shifts the redox balance, contributing to pathological outcomes such as preeclampsia and intrauterine growth restriction (IUGR) with impaired uterine vascular adaptation to pregnancy. Arrows indicate functional outcomes. Catalase (CAT); Glutathione peroxidases (GPx); Hydrogen peroxide (H_2_O_2_); Superoxide (O_2_^•−^); Superoxide dismutases (SOD); Vitamin C (ViT-C); Vitamin E (ViT-E).

During pregnancy, a tightly regulated balance between ROS production and antioxidant defense is essential to ensure proper uterine vascular remodeling, Figure 2. Among the endogenous antioxidant systems, enzymatic antioxidants such as superoxide dismutase (SOD), catalase (CAT), glutathione peroxidases (GPx), glutathione reductase (GSR), and thioredoxin (TRX) play a pivotal role in maintaining redox homeostasis and facilitating physiological vascular adaptations. Oxidation capacity in pregnancy can be improved by aerobic exercise as well as nutraceutical compounds including curcumin–olive oil [50,51]. SOD is responsible for the dismutation of O_2_^•−^ into H_2_O_2_ and exists in three isoforms: SOD1 (cytosolic), SOD2 (mitochondrial), and SOD3 (extracellular). In the context of normal pregnancy, SOD activity increases to support low-to-moderate-level ROS signaling, which is crucial for processes such as angiogenesis, NO bioavailability, and SMC relaxation [52]. ROS can act through several molecular mechanisms including PlGF and potassium calcium channels (K_Ca_s) [53,54]. However, in preeclamptic pregnancies, SOD2 expression is significantly downregulated in the placenta and uterine arteries, contributing to mitochondrial oxidative stress, endothelial dysfunction, and impaired spiral artery remodeling [26]. CAT, another critical antioxidant enzyme, decomposes H_2_O_2_ into H_2_O and O_2_, providing a second layer of protection against H_2_O_2_ accumulation. In pathological pregnancies, such as preeclampsia or IUGR, CAT activity is often decreased, particularly in the syncytiotrophoblasts and decidual ECs, leading to H_2_O_2_ accumulation and redox imbalance [42,55]. Similarly, GPx, especially GPx1 and GPx4, are essential for reducing both H_2_O_2_ and lipid peroxides using glutathione as an electron donor. Impaired GPx expression and activity, as well as polymorphisms in GPx4, have been associated with increased oxidative damage in the placenta and have been implicated in the etiology of preeclampsia [56]. The recycling of oxidized glutathione (GSSG) back to its reduced form (GSH), necessary for GPx function, is catalyzed by glutathione reductase (GSR). Estrogen regulates the GSH/GSSG ratio in the uterine artery, enhances SOD and CAT activity, and, along with decreased NOX4 expression, contributes to the increased uterine artery blood flow during pregnancy [57,58,59]. In preeclamptic pregnancies, decreased GSR activity and glutathione depletion have been observed in maternal serum and placental tissue, compromising the antioxidative capacity and facilitating endothelial oxidative injury [60,61]. Moreover, the TRX system, which contributes to protein redox regulation and modulates redox-sensitive transcription factors such as NF-κB and HIF-1α, is also dysregulated in preeclamptic placentas. Reduced TRX expression has been linked to increased inflammation, trophoblast apoptosis, and impaired placental vascular development [62,63]. Together, these findings support the notion that a functional enzymatic antioxidant defense system is fundamental to maintaining redox signaling in the uterine vasculature. Recent research has explored potential therapeutic strategies aimed at enhancing enzymatic antioxidant defenses. Dietary supplementation with trace elements such as selenium and zinc cofactors for GPx and SOD, respectively, has shown promise in improving placental redox balance in experimental models. Furthermore, pharmacological activation of NRF2, a key transcription factor regulating antioxidant gene expression, may offer a future therapeutic avenue to restore redox homeostasis and support vascular health during complicated pregnancies [64].

### 4.2. Non-Enzymatic Antioxidants

Non-enzymatic antioxidants including glutathione (GSH), vitamins C (ascorbic acid) and E (alpha-tocopherol), and dietary polyphenols form a first line of defense against oxidative stress in the uterine vasculature during pregnancy [3,65], Figure 2. GSH, the most abundant intracellular thiol, functions as the principal redox buffer, detoxifying ROS and regenerating other antioxidants such as vitamins C and E. In healthy pregnancy, high GSH levels in ECs and SMCs support NO bioavailability, angiogenesis, and ECM remodeling, key processes for the appropriate remodeling of uterine arteries. However, preeclamptic and IUGR pregnancies consistently exhibit decreased GSH and GSH/GSSG ratios in both placental and maternal circulation, indicating GSH depletion and impaired redox buffering; this imbalance correlates with endothelial dysfunction and spiral artery remodeling failure [66,67]. Vitamin C and vitamin E act as synergistic, low-molecular-weight antioxidants capable of directly scavenging ROS (e.g., superoxide, lipid peroxides) and regenerating oxidized enzymatic antioxidants. While meta-analyses have reported modest reductions in oxidative stress biomarkers following supplementation during pregnancy, clinical outcomes remain inconsistent for preeclampsia and fetal growth parameters [68,69]. Large-scale randomized controlled trials have failed to show benefits of vitamin C and E supplementation on obstetric outcomes, and some evidence suggests potential pro-oxidant effects at high doses [70].

Dietary polyphenols such as flavonoids and phenolic acids additionally support redox homeostasis by scavenging ROS and upregulating endogenous defense systems. Observational data indicate that higher polyphenol intake is associated with healthier pregnancy outcomes, but robust interventional trials in uterine vascular health remain lacking [71]. Together, these non-enzymatic antioxidants complement enzymatic defenses, maintaining a redox environment conducive to adaptive signaling in uterine arteries. Their insufficiency in pathological pregnancies contributes to oxidative damage, impaired vasodilation, and heightened uterine vascular resistance, exacerbating risks for preeclampsia and IUGR. While micronutrient-rich diets may support vascular health, current evidence does not support routine supplement use; instead, selective strategies to optimize endogenous GSH levels and dietary antioxidant intake warrant further study with rigorous clinical endpoints.

## 5. Inflammatory and Immune-Mediated Mechanisms

### 5.1. Endothelial Activation and Leukocyte Recruitment

During early pregnancy, controlled inflammation is essential for successful uterine vascular remodeling, facilitating trophoblast invasion and spiral artery transformation [72]. However, excessive or dysregulated immune activation can compromise these processes, contributing to obstetric complications such as preeclampsia and IUGR. Pro-inflammatory cytokines, particularly tumor necrosis factor-alpha (TNF-α), interleukin-1 beta (IL-1β), and interleukin-6 (IL-6) secreted by activated decidual immune cells and trophoblasts play a central role in modulating endothelial function. These cytokines upregulate the expression of endothelial adhesion molecules, including vascular cell adhesion molecule-1 (VCAM-1) and intercellular adhesion molecule-1 (ICAM-1), thereby enhancing leukocyte adhesion and transendothelial migration into the decidual and perivascular regions [73]. The recruitment of maternal leukocytes, particularly uNK cells, macrophages, and T cells, is essential in physiological settings, where these cells release angiogenic and matrix remodeling factors. However, in pathological pregnancies, sustained leukocyte–endothelial interactions may promote a chronic inflammatory state, perpetuating endothelial dysfunction [74]. Importantly, TNF-α and IL-1β induce endothelial expression of NADPH oxidase isoforms (NOX1, NOX2), leading to increased generation of ROS [75]. The resulting oxidative stress further amplifies the inflammatory response, creating a feed-forward loop that exacerbates vascular injury. Additionally, pro-inflammatory cytokines suppress the expression of eNOS and impair endothelial barrier integrity, facilitating increased permeability and leukocyte infiltration [76]. These molecular events converge to promote a pro-oxidant, pro-inflammatory endothelial phenotype that hinders the adaptive remodeling of spiral arteries and increases uterine vascular resistance. Moreover, pathological pregnancies such as PE and IUGR are characterized by an altered profile of decidual leukocytes, with a skewed balance toward pro-inflammatory macrophages (M1-like) and Th1/Th17-polarized T cells, which further perpetuate cytokine-driven endothelial activation and oxidative stress [77]. Taken together, these data highlight a critical interplay between inflammation, immune cell trafficking, and redox imbalance in the uterine vasculature.

### 5.2. Decidual Natural Killer (uNK) Cells and Macrophages

**Figure 3:**
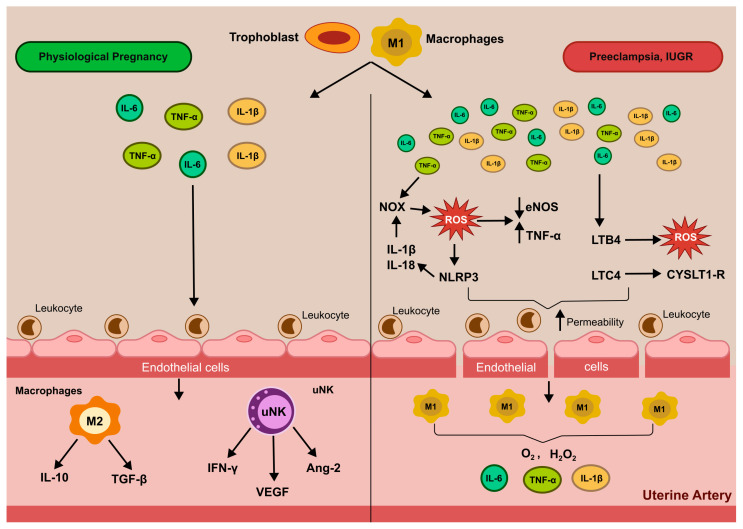
Molecular interplay between trophoblasts and macrophages in uterine artery remodeling during pregnancy. This schematic illustrates how trophoblasts and macrophages coordinate vascular adaptation of the uterine arteries. Both cell types secrete pro-inflammatory cytokines (e.g., IL-1β, TNF-α, IL-6), which induce endothelial adhesion molecules (VCAM-1, ICAM-1) to promote leukocyte adhesion and transmigration. Left (Physiological pregnancy): Decidual natural killer (uNK) cells and M2 (anti-inflammatory) macrophages predominate. uNK cells secrete IFN-γ, VEGF, and angiopoietin-2 to promote immune tolerance and spiral artery transformation. M2 macrophages release TGF-β, support extracellular matrix reorganization, and facilitate vasodilation. Right (Pathological pregnancy: preeclampsia, IUGR): Pro-inflammatory M1 macrophages dominate. The cytokines activate NADPH oxidase (NOX), increasing reactive oxygen species (ROS) production; ROS downregulate eNOS, elevate endothelial permeability, and amplify leukocyte recruitment. M1-derived TNF-α and IL-1β further exacerbate oxidative stress and endothelial dysfunction, impairing uterine artery remodeling. Moreover, trophoblast-derived leukotrienes contribute to endothelial barrier disruption and compromise vascular adaptation. Angiopoietin 2 (Ang-2); Cysteinyl Leukotriene 1 (CysLT1) receptors; Endothelial nitric-oxide synthase (eNOS); Hydrogen peroxide (H_2_O_2_); Interferon-gamma (IFN-γ); Interleukin-6 (IL-6); Interleukin-1 beta (IL-1β); Leukotriene B_4_ (LTB_4_); NADPH oxidase (NOX); Reactive oxygen species (ROS); Superoxide (O_2_^•−^); Transforming growth factor-β (TGF-β); Tumor necrosis factor-alpha (TNF-α); Uterine natural killer (uNK); Vascular endothelial growth factor (VEGF).

Decidual uNK cells and macrophages represent the predominant immune populations within the maternal–fetal interface and are essential regulators of uterine vascular remodeling, Figure 3. uNK cells comprise approximately 70% of decidual leukocytes during the first trimester and are phenotypically distinct from peripheral NK cells, characterized by a CD56^bright CD16^− profile and reduced cytotoxicity [78]. Functionally, uNK cells orchestrate the early stages of spiral artery remodeling through secretion of a range of immunomodulatory and pro-angiogenic factors. These include interferon-gamma (IFN-γ), VEGF, angiopoietin-2 (Ang-2), and PlGF, which act on SMCs and ECs to promote vasodilation, ECM remodeling, and trophoblast-guided invasion [79,80]. IFN-γ, in particular, plays a dual role in mediating vascular transformation and immunological tolerance, ensuring that semi-allogeneic fetal trophoblasts are not rejected [72,81]. Under physiological conditions, uNK are tightly regulated and contribute to signaling processes that support angiogenesis. However, in pathological pregnancies such as preeclampsia or IUGR, oxidative stress may impair uNK cell function.

Decidual macrophages constitute 20–25% of leukocytes at the maternal–fetal interface and are broadly categorized into M1 (classically activated, pro-inflammatory) and M2 (alternatively activated, tissue-repairing, and immunoregulatory) phenotypes. A balanced M1/M2 polarization is crucial for supporting tissue remodeling while preventing excessive inflammation. M2 macrophages, through secretion of IL-10, TGF-β, and MMPs, contribute to the degradation of the ECM and clearance of apoptotic cells, facilitating trophoblast invasion and vascular adaptation [82,83]. In pathological conditions, macrophage polarization may become dysregulated, with a shift toward a pro-inflammatory M1 phenotype leading to oxidative stress. ROS derived from activated macrophages can exacerbate local inflammation and damage to the vascular endothelium, inhibiting NO signaling and reducing vascular compliance [84]. Emerging data also indicate that oxidative stress alters the crosstalk between uNK cells and macrophages, impairing their coordinated contribution to vascular remodeling. For example, increased lipid peroxidation and mitochondrial dysfunction in these immune cells have been reported in preeclamptic placentas, suggesting compromised immune tolerance and reparative functions [85,86]. Overall, the activity of uNK cells and decidual macrophages reflects a delicate immunological balance that integrates inflammatory, oxidative, and angiogenic signals. Disruption of this equilibrium due to oxidative stress contributes to defective spiral artery remodeling and placental hypoperfusion, central features of pregnancy-related vascular disorders.

### 5.3. Inflammasome Activation

The NLRP3 inflammasome is a critical sensor of cellular stress and damage, tightly linked to oxidative stress (OS), sterile inflammation, and vascular dysfunction. Within the uteroplacental environment, the NLRP3 inflammasome is activated in both ECs and trophoblasts in response to diverse danger-associated molecular patterns (DAMPs), including mitochondrial ROS, extracellular ATP, uric acid crystals, and oxidized lipids [8]. Upon activation, NLRP3 oligomerizes and recruits the adaptor protein ASC (apoptosis-associated speck-like protein containing a CARD), which in turn recruits and activates pro-caspase-1. Elevated expression of NLRP3, ASC, and caspase-1 have been consistently reported in placental tissues from preeclamptic pregnancies, correlating with systemic endothelial dysfunction, placental hypoxia, and shallow trophoblast invasion [87,88]. In murine models of preeclampsia, pharmacological inhibition or genetic deletion of NLRP3 mitigates hypertension, improves fetal outcomes, and restores spiral artery remodeling—highlighting a causative role for inflammasome-mediated inflammation in vascular maladaptation [89]. Importantly, oxidative stress serves not only as a trigger but also as a downstream consequence of inflammasome activation. This feedback loop disrupts endothelial function and reduces the capacity for adaptive remodeling of the uterine vasculature, contributing to elevated uterine artery resistance and impaired placental perfusion. Recent studies have also implicated extracellular vesicles (EVs) derived from stressed trophoblasts as carriers of inflammasome components and DAMPs to maternal ECs, facilitating systemic vascular inflammation in preeclampsia [8,90]. This cell–cell communication axis may represent a key mechanism through which localized placental stress translates into widespread maternal endothelial activation. In sum, the NLRP3 inflammasome acts as a central hub at the intersection of oxidative and inflammatory signaling within the uterine microenvironment. Under physiological conditions, its activation is tightly regulated to support immune surveillance and tissue remodeling. However, dysregulation in pathological pregnancies contributes to a pro-inflammatory, pro-oxidant milieu that hinders vascular adaptation, highlighting its potential as a therapeutic target for oxidative stress-related obstetric complications.

### 5.4. Lipid Mediators

Bioactive lipid mediators, including prostaglandins, leukotrienes, and specialized pro-resolving mediators (SPMs), play essential roles in the dynamic regulation of vascular tone, endothelial permeability, and immune responses within the uterine microenvironment during pregnancy. These molecules are derived from the enzymatic oxidation of arachidonic acid and other polyunsaturated fatty acids (PUFAs) via cyclooxygenases (COX), lipoxygenases (LOX), and cytochrome P450 monooxygenases [91]. Among the prostaglandins, prostaglandin E_2_ (PGE_2_) and prostaglandin F_2_α (PGF_2_α) exert opposing effects on vascular reactivity. PGE_2_, via its interaction with EP2 and EP4 receptors on ECs and SMCs, stimulates cAMP production, leading to vasodilation, inhibition of platelet aggregation, and reduced vascular resistance [92]. This pathway supports uterine artery relaxation and contributes to increased uteroplacental perfusion during healthy pregnancy. In contrast, PGF_2_α and thromboxane A_2_ (TXA_2_) signal through thromboxane–prostaglandin (TP) receptors, activating phospholipase C and elevating intracellular Ca^2^⁺ levels, promoting vasoconstriction, vascular smooth muscle contraction, and increased resistance in uterine arteries [93]. An imbalance between vasodilatory [(PGE_2_, and prostacyclin (PGI_2_)] and vasoconstrictive (PGF_2_α, TXA_2_) prostanoids has been observed in pregnancies complicated by preeclampsia and IUGR, where enhanced thromboxane-to-prostacyclin ratios contribute to endothelial dysfunction and impaired arterial remodeling [94].

Leukotrienes, particularly leukotriene B_4_ (LTB_4_) and cysteinyl leukotrienes (LTC_4_, LTD_4_), are also upregulated in placental tissues under inflammatory and oxidative conditions. LTB_4_ enhances neutrophil recruitment and ROS production, while LTC_4_ and LTD_4_ increase endothelial permeability and promote vascular leakage through activation of Cysteinyl Leukotriene 1 (CysLT1) receptors [95,96]. These mechanisms contribute to tissue edema, immune cell infiltration, and oxidative damage within the uterine vasculature. Furthermore, oxidative stress influences lipid mediator synthesis by modifying the activity of COX and LOX enzymes and through peroxidation of membrane phospholipids. Lipid peroxidation products such as 4-hydroxynonenal (4-HNE) and isoprostanes are elevated in the placenta and circulation of preeclamptic women, where they serve both as markers and mediators of vascular injury, promoting endothelial activation and leukocyte adhesion [97]. In recent years, attention has shifted toward specialized pro-resolving mediators (SPMs), including resolvins, protectins, and maresins, which are synthesized from omega-3 PUFAs (e.g., EPA, DHA). These mediators promote the resolution of inflammation by limiting neutrophil infiltration, enhancing macrophage efferocytosis, and restoring endothelial integrity [98]. Although SPMs are present in the decidua and placenta, their levels appear diminished in pathological pregnancies, suggesting a failure to counterbalance pro-inflammatory lipid signaling. Taken together, these findings highlight the pivotal and context-dependent role of lipid mediators in modulating uterine artery remodeling during pregnancy. The balance between pro-inflammatory/pro-constrictive and pro-resolving/vasodilatory lipid signals is tightly linked to oxidative stress and immune activation, underscoring the therapeutic potential of targeting lipid pathways to restore vascular homeostasis in pregnancy complications.

## 6. Crosstalk Between Oxidative Stress and Inflammation

### 6.1. ROS-Mediated Activation of NF-kB

**Figure 4:**
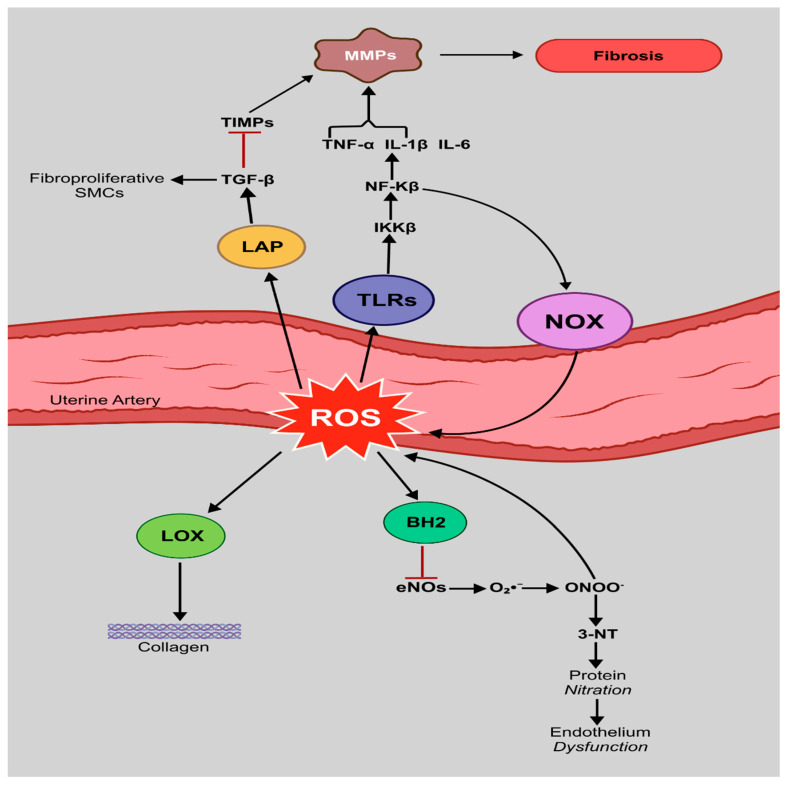
Crosstalk between oxidative stress and inflammation in uterine artery during pregnancy. The schematic illustrates a self-amplifying loop between ROS and pro-inflammatory cytokines that drives oxidative stress and inflammation, leading to endothelial dysfunction and impaired uterine artery remodeling in preeclampsia and IUGR. ROS-mediated NF-κB activation is performed by the IκB kinase β (IKKβ) and upregulating genes for TNF-α, IL-6, IL-1β, and NADPH oxidase, which generates more ROS and reinforces the inflammatory cascade. Further, ROS oxidize the eNOS cofactor tetrahydrobiopterin (BH_4_) to dihydrobiopterin (BH_2_), causing eNOS to produce superoxide (O_2_^•−^) which combines with NO to form peroxynitrite (ONOO^−^), which nitrates key endothelial proteins and disrupts vascular function. Moreover, ROS induce the release of active TGF-β from its latency-associated peptide (LAP). TGF-β promotes fibrosis by (1) inhibiting tissue inhibitors of metalloproteinases (TIMPs); (2) activating matrix metalloproteinases (MMPs), a process further enhanced by IL-1β and TNF-α; and (3) driving smooth muscle cells toward a fibroproliferative phenotype. In addition, ROS boost lysyl oxidase (LOX) activity, which catalyzes collagen crosslink formation. The combined effect of excessive collagen deposition and MMP/TIMP imbalance leads to vascular fibrosis and impaired arterial remodeling. Nuclear factor-kappa B (NF-κB); Reactive oxygen species (ROS); IκB kinase beta (IKKβ); Tumor necrosis factor-alpha (TNF-α); Interleukin-6 (IL-6); Interleukin-1 beta (IL-1β); Nicotinamide adenine dinucleotide phosphate (NADPH); Endothelial nitric oxide synthase (eNOS); Transforming growth factor-beta (TGF-β).

ROS not only serve as damaging agents in oxidative stress but also function as critical signaling molecules that modulate inflammatory pathways. One of the most well-characterized mechanisms through which ROS amplify inflammation is via the activation of the transcription factor NF-κB, Figure 4. This orchestrates the expression of pro-inflammatory and pro-oxidant genes at the maternal–fetal interface. Under homeostatic conditions, NF-κB remains sequestered in the cytoplasm by its inhibitor, IκB. However, upon oxidative stress, ROS stimulate the activation of IκB kinase beta (IKKβ), which phosphorylates IκB, marking it for ubiquitination and proteasomal degradation. The degradation of IκB allows the NF-κB heterodimer (typically p65/p50) to translocate into the nucleus, where it binds to κB motifs on DNA and promotes the transcription of multiple pro-inflammatory genes, including TNF-α, IL-6, IL-1β, COX-2, and inducible nitric oxide synthase (iNOS) [99]. Importantly, NF-κB also induces the expression of NOX subunits, such as NOX1, NOX2, and p22phox, further enhancing ROS production and establishing a feed-forward loop that sustains inflammation and redox imbalance [100]. This self-reinforcing cycle is particularly detrimental in the uterine vasculature, where prolonged NF-κB activation disrupts endothelial homeostasis, promotes leukocyte adhesion, and contributes to impaired spiral artery remodeling. In normal pregnancy, NF-κB activation is transient and spatially regulated, supporting implantation, immune tolerance, and vascular adaptation. However, in pathological conditions such as preeclampsia and IUGR, exaggerated and prolonged NF-κB activity has been observed in placental tissues, maternal circulation, and uterine ECs [101]. Mechanistically, ROS derived from mitochondrial dysfunction, NADPH oxidases, and xanthine oxidase are the primary upstream activators of the NF-κB pathway in the decidua and placental villi. This pathway is further amplified by circulating DAMPs, such as HMGB1 and oxidized lipids, which engage toll-like receptors (TLRs) on endothelial and immune cells, culminating in enhanced IKK activity and NF-κB signaling [102]. The result is a chronic pro-inflammatory state that alters endothelial function, impairs vasodilation via reduced NO bioavailability, and fosters vascular stiffening and hypoperfusion of the uteroplacental unit. In animal models, pharmacologic inhibition of IKKβ or antioxidants that suppress ROS production have been shown to attenuate NF-κB activation, reduce inflammatory cytokine expression, and restore uterine artery remodeling [99,103]. Taken together, the ROS–NF-κB axis represents a central molecular hub in the bidirectional crosstalk between oxidative stress and inflammation. Its dysregulation contributes significantly to endothelial dysfunction and failed vascular remodeling in complicated pregnancies, making it a promising target for therapeutic intervention.

### 6.2. eNOS Uncoupling and Peroxynitrite Formation

eNOS plays a pivotal role in maintaining uterine vascular tone and homeostasis during pregnancy through the production of NO, a vasodilator signaling molecule. Under physiological conditions, eNOS functions as a dimeric enzyme requiring several cofactors, including L-arginine, flavins, and the critical redox-sensitive cofactor tetrahydrobiopterin (BH_4_), to catalyze the conversion of L-arginine to NO [104]. However, in oxidative environments, BH_4_ is rapidly oxidized to dihydrobiopterin (BH_2_), leading to eNOS uncoupling, a pathological state in which the enzyme generates O_2_^•−^ instead of NO [104]. This switch not only diminishes NO bioavailability but also exacerbates oxidative stress by amplifying ROS generation directly within the endothelium. Uncoupled eNOS-derived O_2_^•−^ synergizes with ROS from other sources, particularly NOX, promoting the formation of peroxynitrite (ONOO^−^), a potent nitrating and oxidizing agent [24]. ONOO^−^ reacts with tyrosine residues on proteins, forming 3-nitrotyrosine (3-NT) adducts that disrupt the function of key vascular enzymes and signaling pathways. One notable target is prostacyclin synthase, whose nitration inhibits production of PGI_2_, a vasodilator and inhibitor of platelet aggregation, thereby shifting the balance toward vasoconstriction and thrombosis [105]. Accumulating evidence shows increased 3-NT levels in uterine and placental tissues from women with preeclampsia and IUGR, where they correlate with impaired spiral artery remodeling, reduced uteroplacental blood flow, and systemic endothelial dysfunction [106]. Moreover, ONOO^−^-mediated nitration of mitochondrial enzymes can impair trophoblast energy metabolism and further increase ROS production, establishing a damaging vicious cycle of oxidative and nitrosative stress [107]. In experimental models, pharmacological strategies aimed at restoring BH_4_ levels by supplementation with BH_4_ analogs, folates, or antioxidants like vitamin C have been shown to recouple eNOS, reduce peroxynitrite formation, and improve endothelial function and fetal growth outcomes [104,108].

### 6.3. Matrix Remodeling and Fibrosis

During normal pregnancy, ECM remodeling in the uterine vasculature, particularly in spiral arteries, is essential to reduce vascular resistance and support placental perfusion. This remodeling process involves degradation and reorganization of basement membrane components, SMC dedifferentiation, and appropriate collagen turnover, primarily governed by MMPs and TIMPs, which are modulated by local inflammatory and redox signals [109]. Inflammatory cytokines including IL-1β, TNF-α, and TGF-β induce the transcription and activation of several MMPs, which initially promote ECM degradation but can paradoxically contribute to matrix instability and maladaptive remodeling if persistently upregulated [109]. At the same time, ROS enhance the activation of latent TGF-β by oxidatively modifying the latency-associated peptide (LAP), liberating active TGF-β from the large latent complex. This activated TGF-β acts as a master profibrotic cytokine, driving fibroproliferative transitions in vascular SMCs, increasing synthesis of collagen I/III, fibronectin, and other ECM components [110]. Furthermore, oxidative stress modifies lysyl oxidase (LOX), the enzyme responsible for collagen and elastin cross-linking, through S-nitrosylation, enhancing its enzymatic activity and promoting excessive collagen stabilization and deposition in the vascular wall [111]. The resulting effect is an increase in “passive” stiffness, which reduces arterial compliance and impairs adaptive vasodilation of uterine arteries. Histological studies in preeclamptic placentas and myometrial arteries have shown increased collagen density, altered elastin content, and fibrosis-associated markers such as α-SMA, periostin, and connective tissue growth factor (CTGF), which correlate with increased vascular resistance and poor fetal outcomes [112]. Altogether, these events result in the transition from physiological remodeling to fibrotic stiffening, contributing to noncompliant uterine vessels that are unable to adapt to the hemodynamic demands of pregnancy. Therapeutic strategies that target TGF-β signaling, MMP regulation, or LOX activity—particularly in conjunction with antioxidant approaches—may hold promise in mitigating uterine vascular fibrosis in pregnancy-related disorders.

### 6.4. Translational Evidence (Human Trials)

Across randomized trials in pregnancy, broad antioxidant supplementation has not consistently improved clinical outcomes. A large multicenter trial of high-dose vitamins C/E showed no reduction in pregnancy-associated hypertensive complications [113]. In established severe preeclampsia/HELLP, oral N-acetylcysteine did not stabilize disease or prolong gestation [114]. A single-center trial reported a lower incidence of preeclampsia with coenzyme Q10 in high-risk women, but this finding requires confirmation in larger, more diverse cohorts [115]. Overall, current *human* evidence does not support routine antioxidant supplementation in pregnancy; definitive, biomarker-guided, adequately powered randomized controlled trials are warranted.

## 7. Conclusions

Over the past decade, research has elucidated the dualistic nature of oxidative stress and inflammation in uterine vascular adaptation: as indispensable modulators of angiogenesis and remodeling when tightly regulated, but as drivers of arterial dysfunction and adverse pregnancy outcomes when dysregulated. Key mechanistic insights include the roles of NOX isoforms, mitochondrial ROS, the NLRP3 inflammasome, and eNOS uncoupling in shaping vascular phenotype. Clinical translation is underway through biomarker-guided risk stratification and targeted therapies including low-dose aspirin, statins, and emerging mitochondrial-targeted antioxidants. To fully harness these advances, future work must integrate omics, innovative imaging, and precision trial design, laying the groundwork for personalized maternal–fetal health interventions.

## Supplementary Materials

https://www.mdpi.com/article/10.3390/antiox14091051/s1, Table S1: Summarizing redox/inflammatory biomarkers and their diagnostic/monitoring potential in pregnancy complications. Table S2: Summarizing key oxidative, inflammatory pathways, and associated biomarkers.

## Abbreviations

The following abbreviations are used in this manuscript:

Ang-1/2	Angiopoietin1–2
BH_2_	Dihydrobiopterin
BH_4_	Tetrahydrobiopterin
CAT	Catalase
COX	Cyclooxygenases
CTGF	Connective tissue growth factor
CysLT1	Cysteinyl Leukotriene 1
ECM	Extracellular matrix
ECs	Endothelial Cells
EDCF	Endothelium-derived contracting factors
EDHF	Endothelium-derived hyperpolarizing factor
eNOS	Endothelial nitric oxide synthase
ET-1	Endothelin 1
GPx	Glutathione peroxidases
GSH	Glutathione
GSR	Glutathione reductase
GSSG	Oxidized glutathione
H_2_O_2_	Hydrogen peroxide
HIF-1α	Hypoxia-Inducible Factor 1-alpha
ICAM-1	Intercellular adhesion molecule-1
IFN-γ	Interferon-gamma
IKKβ	IκB kinase beta
IL-1β	Interleukin-1 beta
IL-6	Interleukin-6
iNOS	Inducible nitric oxide synthase
IUGR	Intra-uterine growth restriction
K_Ca_s	Potassium calcium channels
LOX	Lysyl oxidase
LTB_4_	Leukotriene B_4_
MMPs	Matrix-metalloproteinases
NADPH	Nicotinamide adenine dinucleotide phosphate
NF-κB	Nuclear factor-kappa B
NK	Natural killer
NLRP3	NOD-like receptor family pyrin domain-containing 3
NO	Nitric oxide
NOX	NADPH oxidase
NRF2	Nuclear Factor, Erythroid 2-Like 2
3-NT	3-Nitrotyrosine
O_2_^•–^	Superoxide
ONOO^−^	Peroxynitrite
PGE_2_	Prostaglandin E_2_
PGF_2_α	Prostaglandin F_2_α
PGI_2_	Prostacyclin I_2_
PlGF	Placental growth factor
ROS	Reactive oxygen species
sFlt-1	Soluble fms-like tyrosine kinase-1
SMCs	Smooth muscle cells
SOD	Superoxide dismutases
TGF-β	Transforming growth factor-beta
TIMP	Tissue inhibitor of metalloproteinase
TLRs	Toll-like receptors
TNF-α	Tumor necrosis factor-alpha
TP	Thromboxane–prostaglandin
TRX	Thioredoxin
TXA_2_	Thromboxane A_2_
VCAM-1	Vascular cell adhesion molecule-1
VEGF-A	Vascular endothelial growth factor A
XDH	Xanthine dehydrogenase
XO	Xanthine oxidase
