# Supplementary material for: Oxidative Stress and Inflammation in Uterine–Vascular Adaptation During Pregnancy
Source: Antioxidants (Basel). 2025 Aug 26;14(9):1051. doi: 10.3390/antiox14091051 (PMC12466448; doi:10.3390/antiox14091051)
Supplement: Supplementary file 1 [file antioxidants-14-01051-s001.zip › antioxidants-3776694-supplementary.pdf]

Table S1. Summarizing redox/inflammatory biomarkers and their diagnostic/monitoring potential in pregnancy complications.

Biomarker	In preeclampsia/FGR	What it reflects
sFlt-1/PlGF ratio	↑	Anti-angiogenic shift
F_2-isoprostanes	↑	Lipid peroxidation
3-nitrotyrosine	↑	$ONOO^-$-mediated protein nitration
Uric acid	↑	XO activity; anti-angiogenic milieu
MDA/4-HNE adducts	↑	Lipid peroxidation load
oxLDL	↑	Oxidative vascular injury
CRP/IL-6/TNF-α	↑	Systemic inflammation
MnSOD/GPx activity	↓	Antioxidant reserve
Uterine artery Doppler PI	↑	Elevated uterine impedance

Table S2. Summarizing key oxidative, inflammatory pathways, and associated biomarkers.

Pathway Source	Main cellular localization	Physiological role in pregnancy	Pathological shift (preeclampsia/FGR)	Representative biomarkers/readout
NOX1/NOX2 (NADPH oxidases)	ECs, SMCs, leukocytes	Low-level $O_2{\bullet}^-/H_2O_2$ signaling; supports angiogenesis & MMP regulation	Upregulated ROS; ↓NO bioavailability; endothelial dysfunction; ↑uterine artery PI	3-nitrotyrosine; UtA PI
NOX4	ECs; ER/mitochondria	Tonic H_2O_2 promotes VEGF signaling & endothelial stability	Dysregulated output; oxidative stress	H_2O_2-linked oxidation patterns
Mitochondrial ROS	ECs, SMCs, trophoblasts	Redox signals (HIF-1α/NRF2); supports remodeling	Mitochondrial dysfunction; excess ROS; stiffness	8-oxo-dG; mtDNA damage; ↓MnSOD
Xanthine oxidase	Endothelium, placenta	Modest redox tone; NO modulation	XO ↑; uricemia; trophoblast apoptosis	Uric acid; XO activity
NF-κB axis	Multiple	Transient activation for implantation/tolerance	Chronic activation → cytokines; NOX induction	TNF-α/IL-6/IL-1β
Inflammasome (NLRP3)	ECs, trophoblasts	Immune surveillance	IL-1β/IL-18 maturation; endothelial injury	Caspase-1 activity; IL-1β/IL-18
TGF-β/LOX & ECM remodeling	SMCs/ECM	Controlled matrix turnover & compliance	Fibrosis; ↑collagen cross-linking; stiffness	CTGF; periostin; collagen I/III
